# Short-term changes in frequencies of circulating leukocytes associated with narrowband UVB phototherapy in people with clinically isolated syndrome

**DOI:** 10.1038/s41598-019-44488-6

**Published:** 2019-05-28

**Authors:** Stephanie Trend, Anderson P. Jones, Lilian Cha, Matthew N. Cooper, Sian Geldenhuys, Marzena J. Fabis-Pedrini, William M. Carroll, Judith M. Cole, David R. Booth, Robyn M. Lucas, Martyn A. French, Scott N. Byrne, Allan G. Kermode, Prue H. Hart

**Affiliations:** 1 Telethon Kids Institute, University of Western Australia, Perth, WA Australia; 2 Centre for Neuromuscular and Neurological Disorders, Perron Institute for Neurological and Translational Science, University of Western Australia, Sir Charles Gairdner Hospital, Perth, WA Australia; 3 St John of God Dermatology Clinic, St John of God Hospital, Perth, WA Australia; 4 University of Sydney, Faculty of Medicine and Health, Westmead Institute for Medical Research, Westmead, Australia; 5 National Centre for Epidemiology & Population Health, Research School of Population Health, Australian National University, Canberra, ACT Australia; 6 Centre for Ophthalmology and Visual Science, University of Western Australia, Perth, WA Australia; 7 UWA Medical School and School of Biomedical Sciences, University of Western Australia, Perth, WA Australia; 8 Institute for Immunology and Infectious Disease, Murdoch University, Perth, WA Australia

**Keywords:** Autoimmunity, Multiple sclerosis

## Abstract

Clinically isolated syndrome (CIS) is the earliest clinical episode in multiple sclerosis (MS). Low environmental exposure to UV radiation is implicated in risk of developing MS, and therefore, narrowband UVB phototherapy might delay progression to MS in people with CIS. Twenty individuals with CIS were recruited, and half were randomised to receive 24 sessions of narrowband UVB phototherapy over a period of 8 weeks. Here, the effects of narrowband UVB phototherapy on the frequencies of circulating immune cells and immunoglobulin levels after phototherapy are reported. Peripheral blood samples for all participants were collected at baseline, and 1, 2, 3, 6 and 12 months after enrolment. An extensive panel of leukocyte populations, including subsets of T cells, B cells, monocytes, dendritic cells, and natural killer cells were examined in phototherapy-treated and control participants, and immunoglobulin levels measured in serum. There were significant short-term increases in the frequency of naïve B cells, intermediate monocytes, and fraction III FoxP3+ T regulatory cells, and decreases in switched memory B cells and classical monocytes in phototherapy-treated individuals. Since B cells are increasingly targeted by MS therapies, the effects of narrowband UVB phototherapy in people with MS should be investigated further.

## Introduction

UV radiation (UVR) has a number of effects on local and systemic immunity. Evidence from mouse studies shows that exposure to sub-erythemal UVR suppresses immune responses to topically applied experimental antigens that are taken up by Langerhans cells and dermal dendritic cells (DCs) and transferred to draining lymph nodes, inducing the generation of T-regulatory (Treg) cells. This process is assisted by UVR-induced immunoregulatory cytokines, neuropeptides and products of other pathways, including the vitamin D pathway, activated by UVR [reviewed in^1,2^]. Other immunoregulatory cells have also been implicated in UVR-induced immunosuppression, including regulatory B cells (Bregs)^3^, bone marrow-derived DCs^4^ and macrophages^5^, mast cells^6^ and NK cells^7^.

UVR immunoregulation has also been confirmed in humans, with UVR exposure causing reduced responses to antigens applied to both UV-irradiated and non-irradiated skin^2^. However, the mechanisms by which UVR may stimulate systemic immunosuppression in humans, and whether UVR exposure can be used to halt or modulate the progression of an immune-mediated disease such as multiple sclerosis (MS), are less clear. Narrowband UVB delivered to lesional skin is a mainstay of treatment for psoriasis, with UV-induced immunoregulatory circuits thought to operate locally^8^, although systemic effects are also observed. In humans exposed multiple times to sub-erythemal narrowband UVB, there have been varied and contradictory reports of increased numbers of circulating Tregs^9–12^. A reduced frequency of circulating NK cells has also been reported in humans following multiple sub-erythemal UVB exposures^7,13^.

MS is an inflammatory neurological condition, and though a number of genetic and environmental risk factors have been implicated in the onset of MS, fewer factors are known to affect disease activity and progression. However, low lifetime environmental UVR exposure prior to the first evidence of demyelination has been associated with a more rapid transition to MS and more relapses^14^. Since clinically isolated syndrome (CIS) is the earliest clinical episode in the MS pathway, patients with CIS were chosen for participation in a trial of narrowband UVB phototherapy, which aimed to prevent the progression of CIS to MS (the PhoCIS trial). This research group has previously reported that people with CIS, in comparison to healthy controls, have an imbalance in the proportion of suppressive “fraction I” (FrI) and non-suppressive FrIII FoxP3+ Treg cells, together with lower expression of Helios, a transcription factor responsible for stabilising Treg suppressive function^15^. People with CIS also have more transitional B cells, CD141+ myeloid DCs and, if just diagnosed (<14 days), more CD56hi NK cells^15,16^.

The clinical outcomes of the PhoCIS trial were previously reported, with a non-significant reduction in MS observed at 12 months in the phototherapy-treated group compared with controls^17^. The current study investigated whether the frequencies of peripheral blood mononuclear cell (PBMC) subsets or serum immunoglobulins were altered by narrowband UVB phototherapy in the same cohort. PBMCs were collected from participants at the time of their recruitment, and after 1, 2, 3, 6 and 12 months on study. However, since a high proportion of participants commenced disease modifying therapies during follow-up, cellular and immunoglobulin data were analysed to 3 months only. At completion of the phototherapy intervention, there was a significantly higher frequency with phototherapy of naïve B cells, intermediate monocytes, and FrIII Tregs, and a significantly lower frequency of classical monocytes and switched memory (SM) B cells in phototherapy-treated participants compared with controls. These effects on PBMC populations were short-term, and one month after phototherapy was ceased, no significant effects of the intervention were detected.

## Methods

### Study participants

Recruitment for this study was conducted in Perth, Western Australia (32°S). The trial design^18^, CONSORT diagram^17^, and clinical outcomes^17^ have been published elsewhere. Briefly, 20 individuals presenting with CIS within 120 days, and meeting PatyA or PatyB criteria based on magnetic resonance imaging (MRI), were included. The biological and clinical characteristics of the participants at enrolment were similar between groups, except that there were more males in the phototherapy group^17^. If participants did not have serum 25(OH)-vitamin D_3_ [25(OH)D]levels > 80 nmol/L at enrolment, they were supplemented with oral vitamin D (n = 7; two in the phototherapy group and five in the non-phototherapy group)^17^. As previously reported^17^, there were no differences in serum 25(OH)D levels between groups at baseline. Phototherapy, and not extra supplementation, increased serum 25(OH)D levels after 2 and 3 months on study, and this increase was independent of season^17^.

MRI scans and medical reviews were performed at 3, 6, and 12 months. All participants (100%) in the control group converted to MS within 12 months of study enrolment, compared with 7 (70%) in the phototherapy group^17^.

This study was carried out in accordance with the recommendations of the National Health and Medical Research Council of Australia’s National Statement on Ethical Conduct in Human Research. The PhoCIS study protocol was approved by the Bellberry Human Research Ethics committee (2014-02-083) and endorsed by the Human Research Ethics Office of the University of Western Australia (RA/4/1/6796). The trial was registered with the Australian and New Zealand Clinical Trials Registry (ACTRN 12614000185662, registered on 19/02/2014). All participants gave written informed consent in accordance with the Declaration of Helsinki prior to study procedures being performed.

### Phototherapy intervention

Participants with CIS were randomised to receive, or not receive (controls), narrowband UVB phototherapy. Those randomised to the intervention group received narrowband UVB phototherapy three times per week for the first 8 weeks (24 sessions in total). Phototherapy was delivered according to the Dundee protocol, based on patient skin type, as previously described^17,18^.

### Blood sampling

Peripheral venous blood was collected at baseline (B), 1 month (1 M), 2 months (2 M), 3 months (3 M), 6 months (6 M) and 12 months (12 M) post-enrolment. There were 10 participants recruited to each group (phototherapy and control), but one control participant was lost to follow up after one week, and therefore could not be included in this longitudinal study. Although most follow-up samples were collected for the remaining participants, there were a small number of samples not collected. The reasons for missing sample collection included when participants were not able to attend appointments (n = 7 samples missed), withdrawal from the blood collection part of the study due to anxiety (n = 4 samples missed), and relocation to another state following completion of phototherapy (n = 2 samples missed). In total, 101 blood samples were collected (Table 1). There was no significant difference between the two study arms in the number of missing samples at each time point (not shown).

**Table 1:** Number of PBMC samples collected during the study.

Time point	No phototherapy	Phototherapy
Baseline	10 (0)	10 (0)
1 month	4 (0)	9 (0)
2 months	7 (1)^+^	9 (0)
3 months	9 (1)^#^	10 (3)^++*^
6 months	8 (4)^#++*^	9 (3)^+**^
12 months	8 (7)^##+++**^	9 (4)^+***^

The number of samples collected from donors treated within 30 days with disease modifying therapies (DMTs) at that time point are shown in brackets.

Superscript symbols indicate the prescribed DMT for each individual: ^#^fingolimod; ^+^dimethyl fumarate; *natalizumab; the count of symbols indicates the number of participants at that time point taking the DMT.

### Serum 25(OH)D levels

Serum 25(OH)-vitamin D_3_ [25(OH)D] was measured by liquid chromatography tandem mass spectrometry, as previously described^19^. As expected, serum 25(OH)D levels were significantly lower in the winter months, and significantly higher in individuals undergoing phototherapy compared with the controls at 2 M and 3M^17^. Since higher serum 25(OH)D levels were a characteristic of the phototherapy group, collinearity between the at-visit 25(OH)D outcome variable and the phototherapy group indicated that at-visit 25(OH)D level was not an appropriate covariate to include in data models. Therefore, baseline serum 25(OH)D level was instead included as a covariate in all data models.

### Measurement of serum immunoglobulins

We previously reported an association between higher serum immunoglobulin (Ig)G3 levels, or IgG3 as a percent of total IgG, and a rapid conversion from CIS to MS^20^. Therefore, whether phototherapy had any effect on the levels of serum Ig levels was investigated. Total IgG, IgA, IgM, and IgG1–4 subclasses were measured in serum samples collected between baseline and the 6 M time point (n = 81) using cytometric bead arrays, as previously described^20^. These data were used to calculate the absolute concentrations of Ig (µg/mL) and IgG subclasses as a proportion of total IgG (%IgG). All time points when sera were examined had corresponding PBMC samples tested by flow cytometry.

### Participant use of disease modifying therapies

At enrolment, all participants were drug naïve and had not received steroids within the past 30 days. However, within the study period of 12 M, many participants converted from CIS to MS^17^ and began disease modifying therapies (DMTs; Table 1). DMT use was not significantly different between the control and phototherapy groups at the different sampling time points (Table 1). Given the diversity of DMTs prescribed, it was not possible to compare the effects of specific DMTs on cell frequencies. Preliminary attempts to include the use of DMTs within 30 days of the blood collection as an additional covariate in data analyses indicated significant collinearity of DMT use with the treatment variable, particularly during the latter time points of the study. Therefore, blood samples from the 6 M and 12 M time points were excluded from analyses, since the effects of DMTs could not be adequately accounted for. Although a small number of individuals included in analyses had been treated with DMTs at the 2 M and 3 M time points, the majority of samples included in the longitudinal analyses were collected from drug-naïve individuals.

### Flow cytometry for identification of subsets in peripheral blood mononuclear cells

PBMCs were isolated from heparinised blood and extensive cellular phenotyping by flow cytometry performed as previously described^15,16^.

The full list of cell phenotypes investigated are listed in Table 2. T cells, B cells, monocytes, dendritic cells (DCs), natural killer (NK) cells, natural killer T (NKT) cells, and presumed γδT cells were all examined as a percent of total PBMC. In addition, B cell, monocyte, DC and NK cell subsets were examined as previously described^16^.

**Table 2:** Description of cell types examined in phototherapy-treated and control individuals. Cells were quantified by flow cytometry as a frequency of the cell type in the bolded header row above that cell population. An asterisk indicates a cell type that was tested for effects of phototherapy in mixed effects models and linear regression.

PBMCs	T cells	CD4+ T cells	Tregs	CD8+ T cells	B cells	NK cells	DCs	Monocytes
-CD4+ T cells* -CD8+ T cells -B cells -NK cells -Monocytes* -Dendritic cells*	-NKT* -γδT *	-Central memory -Naïve* -Effector memory -Effector* -Tregs -Foxp3+ Tregs* -Follicular Tregs	-Naïve Tregs -Memory Tregs -Helios+FoxP3+ Tregs* -Helios− FoxP3+ Treg* -FrI FoxP3+ Treg* -FrII FoxP3+ Treg* -FrIII FoxP3+ Treg*	-Central memory -Naïve* -Effector memory -Effector	-Plasmablasts -Transitional* -Naïve* -Switched memory* -Non-switched memory* -Double negative	-CD56hiCD16lo* -CD56loCD16hi* -CD56hiCD16int -Mature CD56loCD16hiCD57+* -Immature CD56loCD16hiCD57−*	-Plasmacytoid -CD1c+ (myeloid)* -CD141+ (myeloid)*	-Classical* -Intermediate* -Non-classical*

B cell subsets were examined as a percent of total CD19+ B cells, including CD19+CD20− plasmablasts (CD38+CD27+CD24-IgD−), and the CD19+CD20+ cell subsets including naïve B cells (CD27-IgD+CD38lo/−CD24lo–hi), transitional B cells (CD27-IgD+CD38+ CD24hi), double negative (IgD-CD27−) B cells, non-switched memory (NSM) B cells (CD27+IgD+), and switched memory (SM) B cells (CD27+IgD−)^16^.

Monocyte subsets were examined as a percent of total monocytes, including classical monocytes (CD14+CD16lo/−), intermediate monocytes (CD14+CD16int) and non-classical monocytes (CD14loCD16+)^16^.

DCs subsets were examined as a percent of total DCs, including CD141+ myeloid DCs, CD1c+ myeloid DCs, and CD303+ plasmacytoid DCs (pDCs)^16^.

NK cell subsets were examined as a percent of total NK cells, including CD56hiCD16lo NK cells, CD56hiCD16int NK cells, CD56loCD16hi NK cells, CD56loCD16hiCD57+ NK cells, and CD56loCD16hiCD57- NK cells^16^.

T regulatory cells (Tregs) and follicular Tregs (Tfr) were identified as CD3+CD4+FoxP3+ cells, with the latter also expressing CXCR5. Tregs were further categorised into resting (FrI), activated (FrII) and non-suppressive (FrIII) fractions based on relative expression of FoxP3 and CD45RA, and as Helios+/− as previously described^15^.

**Figure 1:**
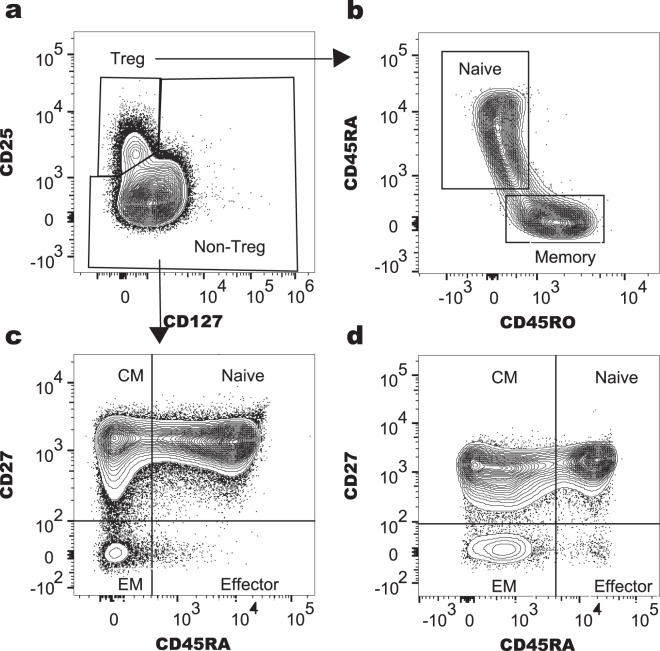
T cell gating strategy applied to data acquired using flow cytometry. T cells from freshly isolated PBMC from study participants were included after gating on PBMC using FSC and SSC, and CD3+ cells. CD3+ cells were differentiated using CD8 versus CD4 plots into CD4+ and CD8+ T cells (not shown). (**a**) CD4+ T cells were separated into Tregs (CD25^+^CD127^lo/neg^) and other cells. (**b**) Tregs identified in panel (a) were further classified as naïve (CD45RA+) or memory phenotypes (CD45RO^+^). (**c**) Non-Tregs identified in panel (a) were classified as central memory (CM) CD4+ T cells (CD27^+^CD45RA^−^), naïve CD4+ T cells (CD27^+^CD45RA^+^), effector memory (EM) CD4+ T cells (CD27^−^CD45RA^−^) and effector CD4+ T cells (CD27-CD45RA+). (**d**) CD8+ T cells were separated into central memory (CM) CD8+ T cells (CD27^+^CD45RA^−^), naïve CD8+ T cells (CD27^+^CD45RA^+^), effector memory (EM) CD8+ T cells (CD27^−^CD45RA^−^) and effector CD8+ T cells (CD27^−^CD45RA^+^).

In addition to our gating panels that were previously published^15,16^, T cells were identified in PBMC by flow cytometry using mouse anti-human surface marker antibodies against CD3 (FITC clone UCHT1), CD4 (APC-H7 clone RPA-T4), CD8 (PE-CF594 clone RPA-T8), CD25 (PE-Cy7 clone M-A251), CD27 (BV421 clone M-T271), CD45RA (APC clone H100), CD45RO (AF700 clone UCHL1), CD127 (BV786 clone HIL-7R-M21), purchased from BD Biosciences (North Ryde, Australia) and data collected using the BD LSR Fortessa flow cytometer and analysed using FlowJo software, as previously described^16^. Tregs were classified according to the traditional definition of CD25+ CD127lo/neg. Traditional Tregs were further characterised as naïve (CD45RA+) or memory (CD45RO+) phenotypes. CD4+ or CD8+ cells were defined as naïve, effector, central memory (CM) or effector memory (EM) T cells, according to the gating strategy shown in Fig. 1.

This approach, which included two different definitions of Tregs (FoxP3+ or traditional CD25+CD127lo Tregs), allowed us to make comparisons of our findings to other reports, that may utilise either of these gating strategies.

### Statistical methods

Differences in categorical outcomes including number of samples affected by DMT use and sex of participants between phototherapy and no phototherapy (control) participant groups at each time point were compared using Fisher’s exact test. Cell frequencies at baseline and at subsequent visits in either phototherapy or control groups were compared using Wilcoxon signed-rank tests. Differences in cell frequencies between groups at specific time points were tested using Mann-Whitney U tests, which showed that the study groups had significantly different frequencies of NKT cells (%PBMC), CD1c+ DCs (%DC), CD141+ DCs (%DC), pDC (%DC), and CD56hiCD16int NK cells (%NK) at baseline. Therefore, all preliminary analyses comparing phototherapy and control participants factored in individual baseline cell frequencies, with data analysed as percent change (Δ) from baseline, using the calculation ((value-baseline)/baseline) × 100.

Given the vast number of cell types measured across many timepoints, an *a priori* structured approach to hypothesis testing was defined to guide the exploratory analysis in the aim of limiting the number of comparisons to be made and reported on. This involved examining the changes in each cell subset over the follow-up period with Mann-Whitney U tests to detect between-group differences or Wilcoxon signed-rank tests to detect time-dependent effects; where a difference was observed (p < 0.1), the basic difference analysis was superseded by the more comprehensive modelling described below (applied to cell phenotypes indicated with an asterisk in Table 2).

Linear mixed effects models with fixed and random effects (random intercept per participant) were used to investigate the longitudinal effects of phototherapy on cell frequencies and serum Ig levels across the follow-up period, controlling for baseline levels as a covariable. All participants with at least one additional sample collected after baseline were included in the analysis. Models were adjusted for age (years), sex, baseline serum 25(OH)D levels (nmol/L), and duration of time on study (days). The 1–2 M mixed effects model incorporated the 1 M and 2 M data, and the 1–3 M mixed effects model incorporated all of the 1 M, 2 M and 3 M data (n = 29 and n = 38 total samples from the control and phototherapy groups, respectively) (Tables 3 and 4).

**Table 3:** Effects of UVB phototherapy on cell frequencies during treatment using data from 1–2 M, adjusted for relevant covariables.

Cell population	Explanatory variable^*^	Estimate	95% Confidence Interval		p-value
			Lower	Upper	
FrIII Treg (%FoxP3 + Treg)	Female sex	4.256	−0.818	9.331	0.096
	Phototherapy	3.619	−0.351	7.588	0.072
	Baseline 25(OH)D	−0.0123	−0.0694	0.0448	0.658
	Time on study	−0.0542	−0.152	0.0436	0.262
	Age	0.0561	−0.183	0.295	0.630
Naïve B cells (%B cells)	Female sex	2.463	−3.64	8.567	0.412
	Phototherapy	8.263	1.80	14.722	**0.015**
	Baseline 25(OH)D	0.128	0.033	0.224	**0.010**
	Time on study	−0.08	−0.26	0.095	0.355
	Age	−0.086	−0.470	0.302	0.652
Switched memory (SM) B cells (%B cells)	Female sex	−0.276	−3.530	2.979	0.862
	Phototherapy	−2.154	−5.481	1.173	0.193
	Baseline 25(OH)D	−0.04	−0.091	0.010	0.110
	Time on study	0.043	−0.048	0.135	0.337
	Age	0.003	−0.196	0.201	0.976
Classical monocytes (%monocytes)	Female sex	−4.056	−9.779	1.667	0.156
	Phototherapy	−7.061	−13.046	−1.075	**0.023**
	Baseline 25(OH)D	0.082	−0.005	0.169	0.064
	Time on study	0.042	−0.114	0.198	0.583
	Age	−0.024	−0.367	0.32	0.888
Intermediate monocytes (%monocytes)	Female sex	1.757	−0.462	3.976	0.115
	Phototherapy	2.903	0.683	5.123	**0.013**
	Baseline 25(OH)D	−0.021	−0.055	0.013	0.222
	Time on study	0.025	−0.036	0.086	0.406
	Age	0.101	−0.036	0.237	0.140

Results are from mixed effect models of longitudinal data. Statistically significant p-values are shown in bolded font.

^*^Data from 1–2 M time points were examined using linear mixed effects models. Phototherapy and sex were included in models as binary outcome variables. Baseline 25(OH)D (nmol/L), time on study (days) and age (years), as well as baseline cell frequency (not shown in Table) were included as continuous variables in the statistical models.

**Table 4:** Effects of UVB phototherapy on cell frequencies both during and at 1 month after treatment, using data from 1–3 M, adjusted for relevant covariables. Results are from mixed effects models using longitudinal data. Statistically significant p-values are shown in bolded font.

Cell population	Explanatory variable*	Estimate	95% Confidence Interval		p-value
			Lower	Upper	
FrIII Treg (%FoxP3 + Treg)	Female sex	1.499	−3.229	6.226	0.525
	Phototherapy	3.760	0.107	7.413	**0.044**
	Baseline 25(OH)D	−0.0567	−0.115	0.00198	0.058
	Time on study	−0.0489	−0.108	0.0105	0.104
	Age	0.115	−0.118	0.347	0.325
Naïve B cells (%B cells)	Female sex	5.254	−0.268	10.776	0.062
	Phototherapy	9.339	3.773	14.905	**0.002**
	Baseline 25(OH)D	0.138	0.051	0.226	**0.003**
	Time on study	−0.071	−0.166	0.023	0.136
	Age	−0.309	−0.659	0.041	0.082
Switched memory (SM) B cells (%B cells)	Female sex	−1.268	−3.754	1.218	0.309
	Phototherapy	−3.175	−5.643	−0.707	**0.013**
	Baseline 25(OH)D	−0.05	−0.089	−0.011	**0.014**
	Time on study	0.031	−0.012	0.074	0.151
	Age	0.015	−0.137	0.167	0.844
Classical monocytes (%monocytes)	Female sex	−3.026	−7.239	1.187	0.155
	Phototherapy	−5.557	−9.92	−1.195	**0.014**
	Baseline 25(OH)D	0.052	−0.014	0.117	0.120
	Time on study	0.034	−0.038	0.105	0.348
	Age	0.016	−0.24	0.271	0.903
Intermediate monocytes (%monocytes)	Female sex	0.931	−1.063	2.925	0.351
	Phototherapy	1.779	−0.183	3.742	0.074
	Baseline 25(OH)D	−0.025	−0.056	0.007	0.120
	Time on study	0.012	−0.022	0.046	0.471
	Age	0.065	−0.058	0.189	0.292

^*^Data from 1– 3 M time points were examined using linear mixed effects models. Phototherapy and sex were included in models as binary outcome variables. Baseline 25(OH)D (nmol/L), time on study (days) and age (years), as well as baseline cell frequency (not shown in Table) were included as continuous variables in the statistical models.

Subsequently, to determine whether phototherapy group differences observed in longitudinal data models occurred at specific study time points, the effects of phototherapy on cell frequencies were investigated at 2 M and 3 M using two separate models of linear regression (ANCOVA framework) (Tables 5 and 6). Model 1 investigated cell frequency differences between groups at each time point adjusting for baseline cell frequency levels as a covariable. A second linear regression model was used (Model 2), adjusting for the baseline levels of cell frequencies as a covariable, as well as age, sex, and baseline serum 25(OH)D levels. The 1 M time point was excluded from linear regression analyses to avoid any bias introduced by the (non-significant) difference in sample numbers between treatment groups at this time point. Where appropriate, models were examined with the inclusion of an indicator term for DMT status, however the inclusion of this term had little impact on the coefficients of interest so models without this term are reported.

**Table 5:** Changes from baseline cell frequencies at the 2 month time point.

Cell population	Explanatory variable*	*Model 1*^			*Model 2* ^#^		
		B	Std. Error	p-value	B	Std. Error	p-value
FrIII Treg (%FoxP3 + Treg)	Phototherapy	2.781	2.154	0.221	4.545	2.88	0.149
	Age				0.179	0.171	0.323
	Sex				−3.624	3.638	0.345
	Baseline 25(OH)D				−0.01	0.042	0.817
Naïve B cells (%B cells)	Phototherapy	11.236	4.129	**0.017**	13.669	4.648	**0.015**
	Age				−0.054	0.309	0.864
	Sex				−5.023	4.670	0.307
	Baseline 25(OH)D				0.157	0.075	0.061
Switched memory (SM) B cells (%B cells)	Phototherapy	−3.806	2.147	0.10	−5.097	2.623	0.081
	Age				−0.019	0.164	0.911
	Sex				2.730	2.624	0.323
	Baseline 25(OH)D				−0.056	0.042	0.21
Classical monocytes (%monocytes)	Phototherapy	−7.248	3.376	0.051	−9.590	4.183	**0.045**
	Age				0.058	0.250	0.821
	Sex				3.925	4.002	0.350
	Baseline 25(OH)D				0.049	0.064	0.459
Intermediate monocytes (%monocytes)	Phototherapy	3.595	1.456	**0.028**	4.114	1.74	**0.040**
	Age				0.122	0.111	0.300
	Sex				−1.661	1.728	0.359
	Baseline 25(OH)D				−0.027	0.027	0.350

Results were obtained using linear regression models. Statistically significant p-values are shown in bolded font.

*Table shows unstandardised coefficients. All models were adjusted for participant’s baseline cell frequencies. ^Model 1 = linear regression for effects of phototherapy (categorical variable) adjusted for baseline cell frequencies. ^#^Model 2 = linear regression adjusted for the baseline levels of cell frequencies as a covariable, as well as age (years), sex, and baseline serum 25(OH)D levels (nmol/L).

**Table 6:** Changes from baseline cell frequencies at the 3 month time point.

Cell population	Explanatory variable*	*Model 1*^			*Model 2* ^#^		
		B	Std. Error	p-value	B	Std. Error	p-value
FrIII Treg (%FoxP3 + Treg)	Phototherapy	5.562	3.078	0.09	4.517	3.417	0.209
	Age				0.239	0.234	0.326
	Sex				2.384	4.468	0.603
	Baseline 25(OH)D				−0.132	0.063	0.056
Naïve B cells (%B cells)	Phototherapy	5.009	5.006	0.332	11.054	5.427	0.063
	Age				−0.685	0.366	0.083
	Sex				−10.311	5.715	0.094
	Baseline 25(OH)D				0.172	0.094	0.090
Switched memory (SM) B cells (%B cells)	Phototherapy	−3.56	1.975	0.09	−4.686	2.169	0.050
	Age				0.053	0.141	0.712
	Sex				2.706	2.272	0.255
	Baseline 25(OH)D				−0.067	0.037	0.096
Classical monocytes (%monocytes)	Phototherapy	−2.844	2.777	0.321	−3.678	3.486	0.311
	Age				0.108	0.212	0.617
	Sex				1.266	3.437	0.719
	Baseline 25(OH)D				−0.001	0.056	0.991
Intermediate monocytes (%monocytes)	Phototherapy	0.38	1.515	0.805	0.189	1.912	0.923
	Age				0.036	0.126	0.777
	Sex				0.178	1.983	0.930
	Baseline 25(OH)D				−0.03	0.033	0.384

Results were obtained using linear regression models. No statistically significant p-values were obtained.

^*^Table shows unstandardised coefficients. All models were adjusted for participant’s baseline cell frequencies. ^Model 1 = linear regression for effects of phototherapy (categorical variable) adjusted for baseline cell frequencies. ^#^Model 2 = linear regression adjusted for the baseline levels of cell frequencies as a covariable, as well as age (years), sex, and baseline serum 25(OH)D levels (nmol/L).

All statistical analyses were performed using SPSS software (v25, IBM corp.), and appropriateness of model fit was determined by visual inspection of diagnostic residual plots. In all analyses, a p-value < 0.05 was considered statistically significant.

## Results

### Effect of narrowband UVB phototherapy treatment on cell frequencies

#### T cell frequencies

There was no association of phototherapy with the frequency of CD4+ or CD8+ T cells, CD4+ Foxp3+ Tregs or Tfr, or CD4+CD25+CD127lo traditional Tregs as a percent of PBMCs (data not shown). There was no association of phototherapy with the proportion of FoxP3+ Tregs expressing Helios.

Phototherapy was associated with a significantly higher frequency of non-suppressive FrIII cells as a proportion of the total CD4+FoxP3+CXCR5− Tregs in the 1–3 M model (Table 4; p = 0.044). Non-significant increases in FrIII cells were associated with phototherapy in the 1–2 M model (Table 3, p = 0.07) and at 3 M in linear regression models prior to adjusting for other variables (p = 0.09) but not after accounting for age, sex, and baseline 25(OH)D levels (p = 0.21) (Table 6).

**Figure 2:**
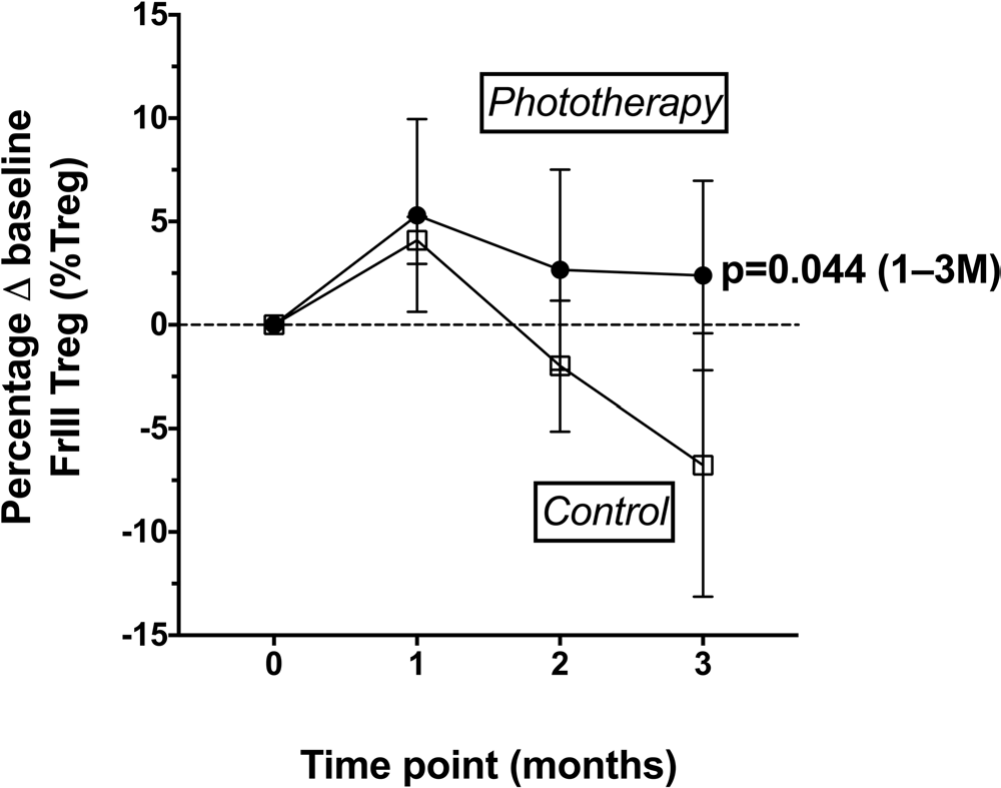
Percent shift from individual baseline frequencies of non-suppressive FrIII Treg cells (%FoxP3 + Tregs). Cells were examined in individuals from control (◻) and phototherapy (•) groups. The group means and SEM are shown. Longitudinal effects of phototherapy detected in mixed effects models including data from 1–3 M are described by text (p = 0.044).

In summary, the frequency of the FrIII Treg population was higher in those who received phototherapy, but no other effects on T cells, particularly immunosuppressive CD4+ Tregs, were observed in this study. The frequencies of FrIII Tregs at each time point as a percent shift from individual baseline frequencies are shown in Fig. 2.

#### B cell frequencies

There were no significant effects of phototherapy on total CD19+ B cell frequencies as a percent of total PBMC. Phototherapy was associated with higher naïve B cell frequencies as a percent of all B cells in both the 1–2 M and 1–3 M models (Tables 3 and 4). Significant differences were observed at 2 M only (in both the adjusted and unadjusted models). By 3 M, the effect of phototherapy was non-significant (p = 0.063) (Tables 5 and 6). These findings together indicate that phototherapy was associated with a short-term effect on the frequency of naïve B cells.

Phototherapy was associated with significantly lower SM B cell frequencies (as a percent of all B cells) in the 1–3 M models (Table 4), but not when including only the 1–2 M data (Table 3). Phototherapy was associated with non-significantly lower SM B cell frequencies at 2 M (Table 5) and 3M (Table 6).

**Figure 3:**
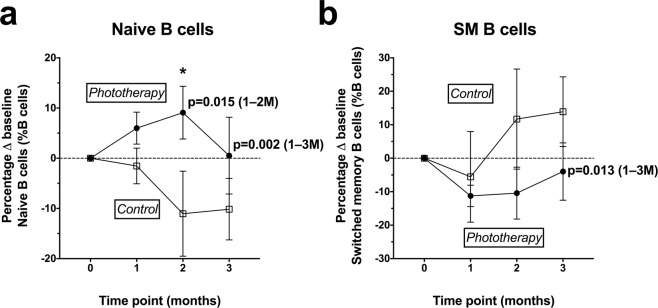
Percent shift from individual baseline cell frequencies of (**a**) naïve, and (**b**) switched memory (SM) B cell subsets, measured in individuals from control (◻) and phototherapy (•) groups. The group means and SEM are shown. Statistical outcomes from linear regression are shown above the 2 M time point where relevant, where * indicates a significant difference between the control and phototherapy groups at that time point (p < 0.05) in at least one data model. Longitudinal effects of phototherapy treatment detected in mixed effects models including data from 1–2 M and from 1–3 M are shown, with significant p-values and the sample time points included described by text.

The frequencies of the B cell populations that were found to be significantly different between treatment groups in mixed effects models are shown in Fig. 3.

In summary, phototherapy was associated with significantly higher naïve B cell frequencies and significantly lower SM B cell frequencies, but these effects were not detected at the 3 M time point (one month after phototherapy treatment had completed).

#### Monocyte frequencies

There was no significant association between phototherapy and total monocyte frequencies as a percent of total PBMC. Phototherapy was associated with a significantly lower frequency of classical monocytes as a percent of monocytes in the 1–2 M and 1–3 M models (Tables 3 and 4).

Phototherapy was also associated with a significantly higher frequency of intermediate monocytes in the 1–2 M phototherapy treatment period (Table 3), but this effect was not significant in the 1–3 M model.

The short-term effects of phototherapy on monocyte subset frequencies were confirmed by linear regression models at 2 M, which showed that classical monocyte frequency was significantly lower, and intermediate monocyte frequency significantly higher, in phototherapy-treated individuals at this time point (Table 6). However, these effects were not present in the 3 M assessment.

**Figure 4:**
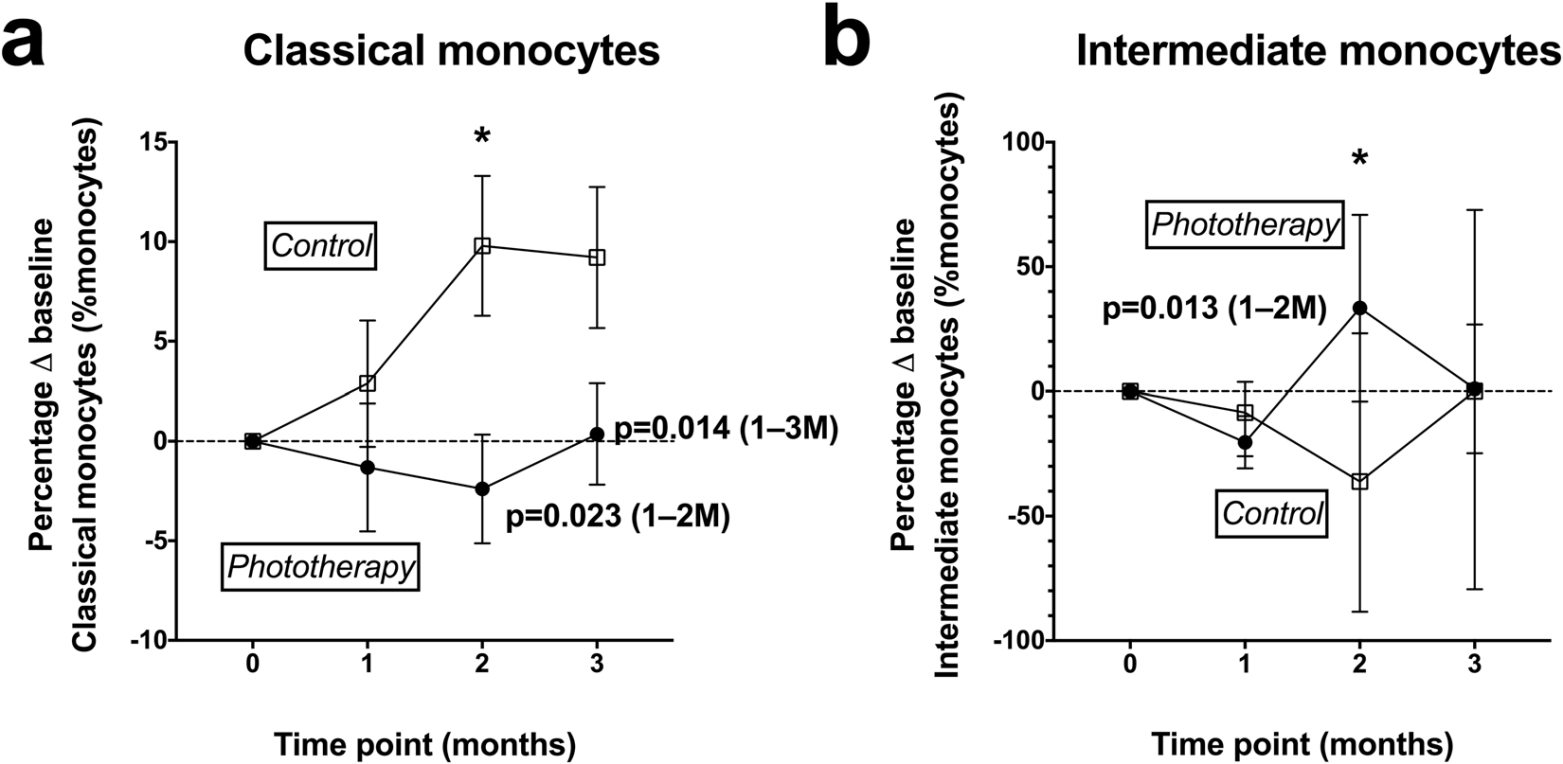
Percent shift from individual baseline cell frequencies of (**a**) classical and (**b**) intermediate monocyte subsets, measured in individuals from control (◻) and phototherapy (•) groups. The group means and SEM are shown. Statistical outcomes from linear regression are shown above the 2 M time point where relevant, where * indicates a significant difference between the control and phototherapy groups at that time point (p < 0.05) in at least one data model. Longitudinal effects of phototherapy detected in mixed effects models including data from 1–2 M and from 1–3 M are shown, with significant p-values and the sample time points included described by text.

In summary, phototherapy was associated with lower classical monocyte frequencies compared to controls and higher frequencies of intermediate monocytes, but only during the phototherapy treatment period. The change from individual baseline cell frequency for classical and intermediate monocytes are shown in Fig. 4.

#### NK cell frequencies

Phototherapy was not associated with changes in total NK cell frequencies as a percent of total PBMC, or subset composition as a percent of NK cells in mixed effects models or linear regression models at any time point.

#### DC frequencies

The frequency of total DCs as a percent of total PBMC, or DC subsets as a percent of total DC were not significantly different between control and phototherapy groups.

### Effects of phototherapy on serum Ig levels

There were no significant effects of phototherapy on the serum levels of total IgG, IgA, IgM, or IgG1–4 subclasses, or the proportions of IgG1–4 as a percent of total IgG in either 1–2 M or 1–3 M mixed effects models (data not shown).

## Discussion

The PhoCIS trial aimed to harness the immunoregulatory effects of narrowband UVB radiation to modulate the disease course in individuals with high-risk CIS^18^. In this study, UVB phototherapy was associated with significantly higher circulating frequency of naïve B cells as a percent of B cells, intermediate monocytes as a percent of monocytes, and non-suppressive FrIII Tregs as a percent of Tregs, and significantly lower frequencies of SM B cells as a percent of B cells and classical monocytes as a percent of monocytes, compared with controls. The effects of the eight weeks of phototherapy were short-lived, and when examined at specific time points using linear regression, effects of phototherapy were observed at the 2 M sample collection point, but not at the 3 M collection point.

The narrowband UVB intervention was associated with significant changes in the frequencies of naïve and SM B cells in the UVB-irradiated individuals, whose levels after treatment were significantly higher and lower, respectively, compared with the controls. To our knowledge, this is the first report of such effects on these B cell subsets in human blood. Although this is the first trial of narrowband UVB phototherapy for CIS, in people with psoriasis for whom this treatment has been a mainstay, UVB exposure decreases the levels in skin of inflammatory cytokines such as TNF-α, IFN-γ, and IL-17, that may influence B cell maturation^21–23^. Although we have not investigated cellular function here, in general, naïve B cells are less responsive to signals that stimulate cell proliferation, Ig secretion and survival than memory B cells due to differentially expressed immunomodulatory receptors such as CD84 and TLRs^24^. In people with MS, peripheral blood naïve B cell frequencies are significantly increased during remission^25^ and upon effective disease treatment^26^, and naïve B cells from people with MS produce more IL-10 than memory B cells^27^. It is possible that IL-10 producing Bregs, which protect against EAE in the mouse model of MS^28^, and are induced by UVR in mice^29,30^, expand within the naïve B cell subset during phototherapy. Further investigation of the B cell compartment may reveal whether the functional activities or survival and/or proliferation of specific B cell subsets are altered by phototherapy in CIS. Effects on the naïve and/or SM B cell populations are also reported for DMTs in MS^26,31,32^. Given the proposed roles of B cells in contributing to MS progression through both antibody-dependent and -independent effects^33,34^, this study suggests that the reduction in frequencies of memory B cells associated with narrowband UVB phototherapy could have benefits for people with CIS.

There were lower frequencies of classical monocytes (CD14+CD16−) after phototherapy compared with control participants at 1–2 M and 1–3 M in mixed effects models, and at 2 M specifically using linear regression. In addition, there were higher intermediate monocyte frequencies (CD14+CD16int) from 1–2 M in mixed effects models and at 2 M using linear regression in phototherapy-treated individuals compared with controls. These findings support a previous report that monocyte expression of CD16 increased following repeated UVB exposure in healthy donors^35^. In people with untreated MS, CD16+ monocytes were reported to have higher expression of a number of activating receptors, including the FcγRI (CD64), and secrete less IL-10 upon *ex vivo* activation compared with classical monocytes^36^. However, following *in vivo* or *in vitro* exposure of monocytes to DMTs, the CD16int monocyte population is reported to expand, produce the most IL-10, and increase phagocytic activity^37,38^, becoming functionally similar to intermediate monocytes in healthy individuals^39^. Therefore, although increased non-classical monocyte frequencies (CD14loCD16+) are a biomarker of active MS disease^40^, intermediate monocytes may actively contribute to suppressing inflammation following successful treatment of MS. Consequently, the higher intermediate monocyte frequency observed after phototherapy in this study suggests potential clinical benefits in MS, and as such, functional assays or additional markers on monocytes should be investigated in future.

UVR-induced increases in Tregs and/or FoxP3 expression by Tregs have been reported in multiple studies in healthy individuals, and those with skin conditions^9,11,12^. However, in the current study, no increase in circulating FoxP3+ or traditional CD4+CD25+CD127lo Tregs was detected in association with phototherapy. In one prior trial of narrowband UVB phototherapy in patients with skin disease, the frequency of circulating FoxP3+ Tregs increased in correlation with serum 25(OH)D levels, up to approximately 50 nmol/L^9^. The individuals in our study had much higher serum 25(OH)D levels at the time of their enrolment, and therefore, it could be speculated that in our participants, UV-inducible expansion of FoxP3+ Tregs had already plateaued. On the other hand, the absence of a UVB-induced expansion of Tregs in the current study and in a prior study of vitamin D-deficient MS patients^10^ suggests a disease-specific inability to induce FoxP3+ Tregs following UVB exposure and/or increased serum 25(OH)D levels. Although phototherapy was not associated with changes in the proportions of FoxP3+ Tregs expressing Helios in the present study, our finding of a proportional increase in the FrIII Treg fraction is consistent with the previously reported UVB-induced expansion in the proportions of Helios- Tregs in people with MS^10^, since FrIII has the highest proportion of Helios- cells^15^. FrIII Tregs exhibit pro-inflammatory rather than suppressive activity compared with FrI and FrII Tregs, and FrIII contains the greatest proportion of CCR6+ T helper (Th)17-like and CCR6+CXCR3+ Th17.1-like Tregs^15,41^. Preferential transmigration of the latter cells into the central nervous system (CNS) in patients with CIS reduces the frequency of these cells in the circulation and is associated with more rapid conversion from CIS to clinically definite MS^42^. Therefore, it is possible that increased frequencies of FrIII Tregs following phototherapy in this study were a result of decreased migration of these cells into the CNS, although no corresponding CNS samples are available to investigate this hypothesis in these participants.

No effect of phototherapy on DCs or NK cells were detected in this study. Narrowband UVB therapy over four weeks in patients with psoriasis was previously associated with decreased frequencies of circulating CD56 + NK cells^7^, and another study found that the numbers of CD1c- DC decrease in the skin after UVB treatment^21^. It is possible that the small sample size available limited our power to detect changes in some cell types. Despite this, changes that were detected in cell populations associated with phototherapy were often repeated in independent statistical analyses, with biological findings reflecting the expected pattern over time, where the largest difference between treatment groups was at the completion of phototherapy at the 2 month time point. Therefore, despite the need to recognise the limitations associated with the small sample size^43^, the conclusions regarding cells changed by phototherapy are logically and statistically supported by the data available. However, more studies are needed to determine the precise effect size of circulating cellular responses to phototherapy, and other potential effects on cell subsets that we may not have detected.

In this study, cellular profiles were examined in blood samples from participants in the PhoCIS trial for which the clinical outcomes have been previously published^17^. In summary, by 12 M, three of ten participants receiving narrowband UVB phototherapy had not converted from CIS to MS whereas all nine participants who did not receive phototherapy converted. In this report, we have pooled changes in cell phenotypes from all ten participants receiving narrowband UVB phototherapy, and all nine not receiving phototherapy. For the three participants who had not converted to MS by 12 M, a longitudinal analysis of their cells from baseline to 3 M was also performed as any consistent changes may help us determine the changes potentially responsible for the beneficial effects of narrowband UVB phototherapy. Although we examined the pattern of cell changes in these three individuals, and one of them had the largest increase in naïve B cells of the cohort after phototherapy, there was no clear and consistent pattern of cell changes in blood from this group of three compared with the individuals who received phototherapy but who converted to MS. Furthermore, there were not enough participants to make valid statistical comparisons between the converters and non-converters, but this could be investigated in future in a larger trial.

Inter-individual differences in baseline serum 25(OH)D levels were accounted for in the analyses presented here. Higher baseline serum 25(OH)D was associated with increased naïve B cell frequency in multiple analyses, and lower SM B cell frequency in the 1–3 M mixed effects model. The effect of baseline 25(OH)D was detected regardless of phototherapy, although for naïve B cells and SM B cells, the effect of phototherapy was larger than that of 25(OH)D (estimates of effect sizes as shown in Tables 3 and 4). All individuals had clinically sufficient levels of 25(OH)D at the time of baseline blood sample collection, and there was no difference between groups in serum 25(OH)D at baseline^17^. *In vitro*, a number of effects of 1,25(OH)_2_-vitamin D_3_ on B cells have been observed^44^, including inhibition of memory B cell differentiation from naïve cells^45^. However, in two previous clinical trials of high dose vitamin D supplementation (≥10,000 IU/day) in people with MS, no effects on naïve B or other B cell frequencies were observed^46,47^, but in the study of Sotirchos *et al*., wherein participants received 20,000 IU/day for 6 months, reductions in IL-17+ and effector memory CD4+ T cells were reported after the intervention^47^. The scale of the effect of phototherapy on B cell frequencies relative to baseline 25(OH)D, particularly when all participants had clinically sufficient serum 25(OH)D levels, suggest that vitamin D-independent effects contributed to the effects on B cell frequencies observed here. However, this study was not designed to resolve whether effects of narrowband UVB phototherapy on PBMC were independent of vitamin D specifically.

In summary, this study expands upon the known biological effects of low dose UVB radiation in humans, and provides data on the short-term effects of narrowband UVB treatment on a new patient population, namely, people with high-risk CIS. Despite an earlier focus on the contribution of Tregs to MS disease, no phototherapy-associated effects on the frequencies of Tregs were detected, and the largest effects associated with phototherapy were detected in naïve B cells. B cells are increasingly recognised as playing important roles in the pathogenesis of MS and recovery from episodes of demyelination^3,26,31,33,44,48^. These findings suggest that narrowband UVB phototherapy may have positive, albeit short-lived, clinical effects on people with MS, and should be investigated further.

## Data Availability

The datasets generated during the current study are available from the corresponding author on reasonable request.
